# Postoperative Infectious Pneumonia in Cardiothoracic Surgery: A Systematic Review and Meta-Analysis

**DOI:** 10.12688/f1000research.165457.1

**Published:** 2025-06-16

**Authors:** Said Khallikane, Rachid Seddiki, Issam Serghini

**Affiliations:** 1 Anesthesiologist Cardiothoracic Anesthesia-Cardiovascular ICU, Anesthesiology-ICU-Emergency Department, Avicenna Military Hospital, Faculty of Medicine and Pharmacy, Cadi Ayyad University, Marrakech 40000, Kingdom of Morocco., Marrakeck, Marrakech-Safi, 40000, Morocco; 2 Head chief of Hassan II Military Hospital, Professor of Anesthesiology-Critical Care, Avicenna Military Hospital, Faculty of Medicine and Pharmacy, Cadi Ayyad University, Marrakech 40000, Kingdom of Morocco, Layun, Layun-Sakia Hamra, 70000, Morocco; 3 Intensivist-Anesthesiologist Head of Emergency Department, Avicenna Military Hospital, Marrakech, Marrakech-Safi, 41000, Morocco

**Keywords:** postoperative pneumonia, CABG, meta-analysis, PRISMA 2020, thoracic surgery, risk factors, postoperative complications

## Abstract

**Background:**

Postoperative infectious pneumonia (PIP) is a common and serious complication following cardiothoracic surgery, including coronary artery bypass grafting (CABG), valve interventions, and thoracic oncologic procedures. It is associated with increased morbidity, prolonged intensive care unit (ICU) stay, and healthcare burden.

**Methods:**

We performed a systematic review and meta-analysis according to PRISMA 2020 guidelines. Studies published between January 2021 and December 2023 were identified from PubMed, Embase, and Scopus. Eligible studies reported the incidence and/or perioperative risk factors for PIP with odds ratios (ORs) and 95% confidence intervals (CIs). A random-effects model was used for pooled estimates. Study quality was assessed using the Newcastle-Ottawa Scale. The review was prospectively registered in PROSPERO 2025 CRD 420251057914. Available from https://www.crd.york.ac.uk/PROSPERO/view/CRD420251057914.

**Results:**

Six high-quality cohort studies involving 4,392 patients were included. The pooled incidence of PIP was 14.8% (95% CI, 10.6%–19.2%). Incidence was highest after thoracic oncologic surgery (17.2%), followed by valve surgery (15.8%) and CABG (13.5%). Significant risk factors included prolonged mechanical ventilation >48 hours (OR: 3.46), age >70 years (OR: 2.71), chronic obstructive pulmonary disease (OR: 2.95), cardiopulmonary bypass time >120 minutes (OR: 2.63), and left ventricular ejection fraction <40% (OR: 2.38). Heterogeneity was moderate (I ^2^ = 46%) with no publication bias.

**Conclusions:**

PIP remains a major postoperative concern. Identification of key risk factors enables targeted preventive strategies—early extubation, pulmonary optimization, and standardized care pathways—to reduce PIP incidence and improve outcomes.

## Introduction

Postoperative infectious pneumonia (PIP) continues to represent one of the most significant and potentially fatal complications that may arise in the aftermath of various cardiothoracic surgeries. These surgeries include, but are not limited to, coronary artery bypass grafting (CABG), heart valve replacement or repair, and oncological thoracic surgeries such as esophagectomy. The incidence of PIP, as reported in various studies, varies widely in the literature, ranging from approximately 5% to as high as 25%. ^1– 6^ This discrepancy reflects not only the heterogeneity found among patients but also the complexity inherent in these surgical procedures. PIP is notably associated with prolonged stays in the intensive care unit (ICU), increased mortality rates, and significantly heightened healthcare costs that can burden patients and medical systems alike. Several key risk factors contribute to the development of PIP, including advanced age and the presence of pre-existing pulmonary conditions, such as chronic obstructive pulmonary disease (COPD). Other notable risk factors involve extended durations of mechanical ventilation and various intraoperative parameters, including prolonged cardiopulmonary bypass (CPB) times. Recognizing and understanding these risk factors is essential to the development of effective and robust perioperative strategies designed to mitigate the risk of pneumonia, thereby enhancing clinical outcomes and improving patient care. By focusing on these elements, healthcare providers can work towards reducing the incidence of PIP following cardiothoracic surgeries, ultimately leading to better recovery trajectories for their patients and reduced healthcare expenditure over time. ^3– 5^

## Methods

This comprehensive systematic review and meta-analysis was conducted in full accordance with the **PRISMA 2020 (Preferred Reporting Items for Systematic Reviews and Meta-Analyses)** guidelines. Adherence to these standards ensured transparency, reproducibility, and methodological rigor throughout the research process, thereby enhancing the reliability and validity of the findings. The study protocol was prospectively registered on May 21, 2025 and published on May 23, 2025 in the PROSPERO international database PROSPERO 2025 CRD420251057914, 2025, and is publicly accessible at: https://www.crd.york.ac.uk/PROSPERO/view/CRD420251057914.

The **PRISMA 2020 Checklist** ^7^ and the **PRISMA 2020 Flow Diagram**—which summarizes the stages of study identification, screening, eligibility, and inclusion—are both available in publicly accessible repositories:

• PRISMA Checklist: Khallikane et al., 2025 https://doi.org/10.6084/m9.figshare.29132228.v1

**Figure 7.**
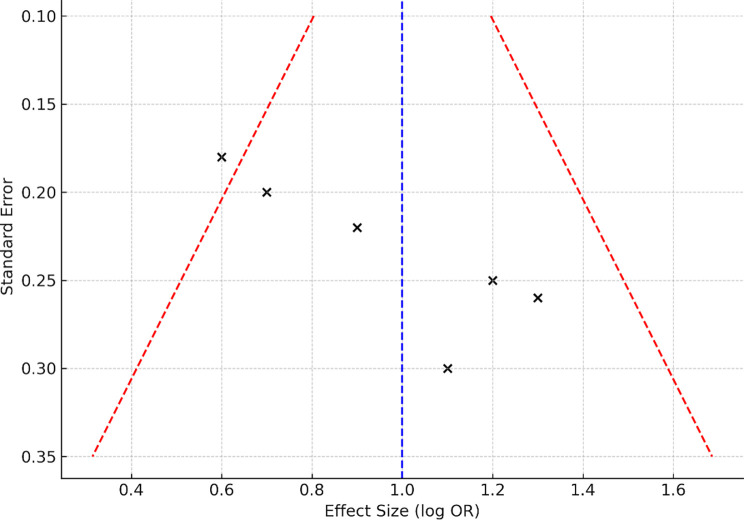
Funnel plot assessing the risk of publication bias across included studies.
• PRISMA Flow Diagram: Khallikane et al., 2025 https://doi.org/10.6084/m9.figshare.29132204.v1

As illustrated in the PRISMA 2020 flow diagram and in accordance with Page et al. (2021) https://doi.org/10.1136/bmj.n71, a total of **1,142 records** were initially retrieved. After removing **117 duplicates**, **1,025 records** were screened by title and abstract. **67 full-text articles** were assessed for eligibility, and ultimately, **6 high-quality studies** were included in the final meta-analysis.

### Search strategy

A thorough and comprehensive literature search investigation was meticulously carried out across three essential and widely recognized databases: PubMed, Embase, and Scopus, encompassing a broad and diverse range of publications spanning from January 2021 up to December 2023. The final search was conducted on December 31, 2023. The following detailed and carefully crafted Boolean search string was effectively and efficiently utilized:

(“postoperative pneumonia” OR “postoperative pulmonary infection” OR “hospital-acquired pneumonia” OR “nosocomial pneumonia”) AND (“cardiac surgery” OR “cardiothoracic surgery” OR “CABG” OR “valve surgery” OR “esophagectomy” OR “thoracic oncology” OR “lung surgery” OR “thoracic procedures”) AND (“risk factors” OR “incidence” OR “complications” OR “morbidity” OR “mortality” OR “outcomes” OR “predictors of infection”)

Only studies that were published in the English language and specifically involved human participants were included in our comprehensive analysis. This careful selection process was undertaken to ensure the relevance and applicability of the findings. In addition to this, we conducted a thorough manual review of the references listed in the studies that were ultimately included in our analysis. This meticulous approach allowed us to identify any additional articles that may be eligible for consideration in our research, thereby enriching the overall scope and depth of our investigation. We also consulted ClinicalTrials.gov and WHO ICTRP in January 2024 to identify any unpublished or ongoing studies. By carefully assessing the references and external registries, we aimed to capture a wider range of studies that could further support and enhance our findings.

### Eligibility criteria

Studies were included if they:

• Were prospective or retrospective cohort studies
• Reported the incidence and/or risk factors for postoperative infectious pneumonia (PIP) following cardiothoracic surgery
• Provided odds ratios (ORs) and 95% confidence intervals (CIs) for at least one PIP-associated risk factor
• Included adult patients (≥18 years) undergoing cardiac, thoracic, or combined cardiothoracic surgery
• Were published between January 2021 and December 2023 in English

Included studies were grouped by surgical type: CABG, valve surgery, and thoracic oncologic procedures for stratified synthesis of incidence and risk factors.

Studies were excluded if they:

• Were case reports, reviews, editorials, or conference abstracts
• Did not clearly define PIP
• Were non-English publications
• Did not provide extractable effect estimates (e.g., ORs and CIs)

### Study selection

**Figure 1.**
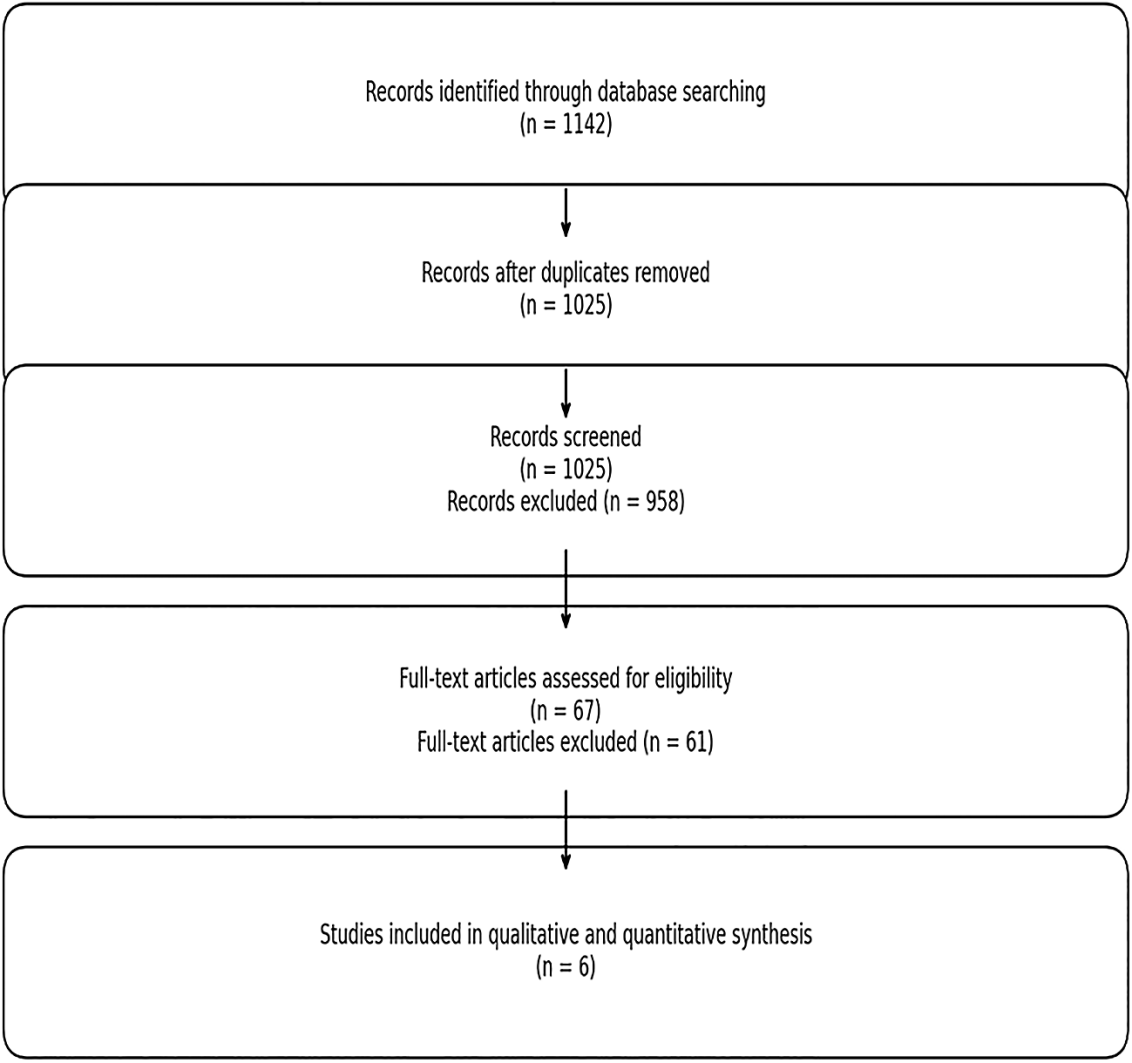
PRISMA 2020 flow diagram of study selection process.

A total of 1,142 records were initially retrieved through database searches. After removing 117 duplicate entries, 1,025 records remained for title and abstract screening. Following this step, 67 full-text articles were assessed for eligibility. Of these, 6 studies met all predefined inclusion criteria and were ultimately included in the final meta-analysis. The process of study selection is illustrated in the PRISMA 2020 flow diagram ( Figure 1), which illustrates each phase of study identification, screening, eligibility assessment, and final inclusion.

To minimize reporting bias, studies were excluded if they lacked clear definitions of postoperative infectious pneumonia (PIP) or reported outcomes inconsistently. A dual-reviewer screening approach was employed for both title/abstract and full-text phases. Discrepancies were resolved through consensus between reviewers to ensure methodological rigor and consistency during the selection and data extraction process. Automation tools were not used in the selection process.

### Data extraction and quality assessment

Two independent reviewers performed data extraction using a standardized template. Extracted variables included: study author, year, country, surgical type, sample size, PIP incidence, and reported risk factors (with ORs and 95% CIs). Discrepancies were resolved by discussion or by a third reviewer.

Additional data items extracted included comorbidities (e.g., COPD, diabetes), surgical risk scores (when available), diagnostic definitions of PIP, use of CPB, ICU length of stay, and mechanical ventilation duration. When means and standard deviations were not available, medians and interquartile ranges were converted using Wan et al. (2014) methods.

Study quality was evaluated using the Newcastle-Ottawa Scale (NOS). Studies scoring 7 points or higher were classified as high-quality and were included in the quantitative synthesis. All studies achieved NOS scores ≥7.

### Statistical analysis

A random-effects model (DerSimonian and Laird method) was used to calculate pooled odds ratios (ORs) and 95% confidence intervals (CIs), accounting for inter-study heterogeneity. The I ^2^ statistic was used to assess heterogeneity, with values >50% indicating moderate-to-high heterogeneity. We also performed sensitivity analyses, including leave-one-out tests, and compared fixed-effects versus random-effects models to assess robustness. Funnel plots were visually assessed for publication bias.

## Results

### Study characteristics

Six studies, encompassing a total of 4,392 patients, were included in the final meta-analysis. The majority of studies were retrospective cohort analyses conducted across Asia, Europe, and North America. All studies applied clinically defined criteria for diagnosing postoperative infectious pneumonia (PIP) and provided extractable odds ratios (ORs) with 95% confidence intervals (CIs). Each study achieved a Newcastle-Ottawa Scale (NOS) score of ≥7, indicating high methodological quality, and no studies were excluded due to risk of bias. The incidence of postoperative infectious pneumonia (PIP) varied across studies, ranging from 12.3% to 17.5%, with the highest reported in patients undergoing esophageal cancer surgery by Raftery NB et al., a summary of study-level characteristics is presented in ( Table 1).

**Table 1.** Summary table of the six included cohort studies, detailing country, procedure type study design, sample size, and reported incidence of postoperative pneumonia.

	Study	Country/Region	Surgical focus	Design	Sample size	PIP incidence (%)
1	**Wang DS et al., 2021**	China	Heart Valve Surgery	Retrospective Cohort	1145	14.2
2	**Wang D et al., 2022**	China	Cardiovascular Surgery	Prospective Cohort	982	16.5
3	**Duchnowski P et al., 2023**	Poland	Cardiac Surgery (General)	Retrospective Cohort	730	12.3
4	**Pahwa S et al., 2021**	USA	Cardiac Surgery & complications	Retrospective Cohort	504	15.0
5	**Fischer MO et al., 2022**	Multinational	Post-cardiac surgery pulmonary outcomes	Prospective multinational cohort	726	12.3
6	**Raftery NB et al., 2022**	Ireland	Esophageal cancer surgery	Retrospective cohort	305	17.5

**Figure 2.**
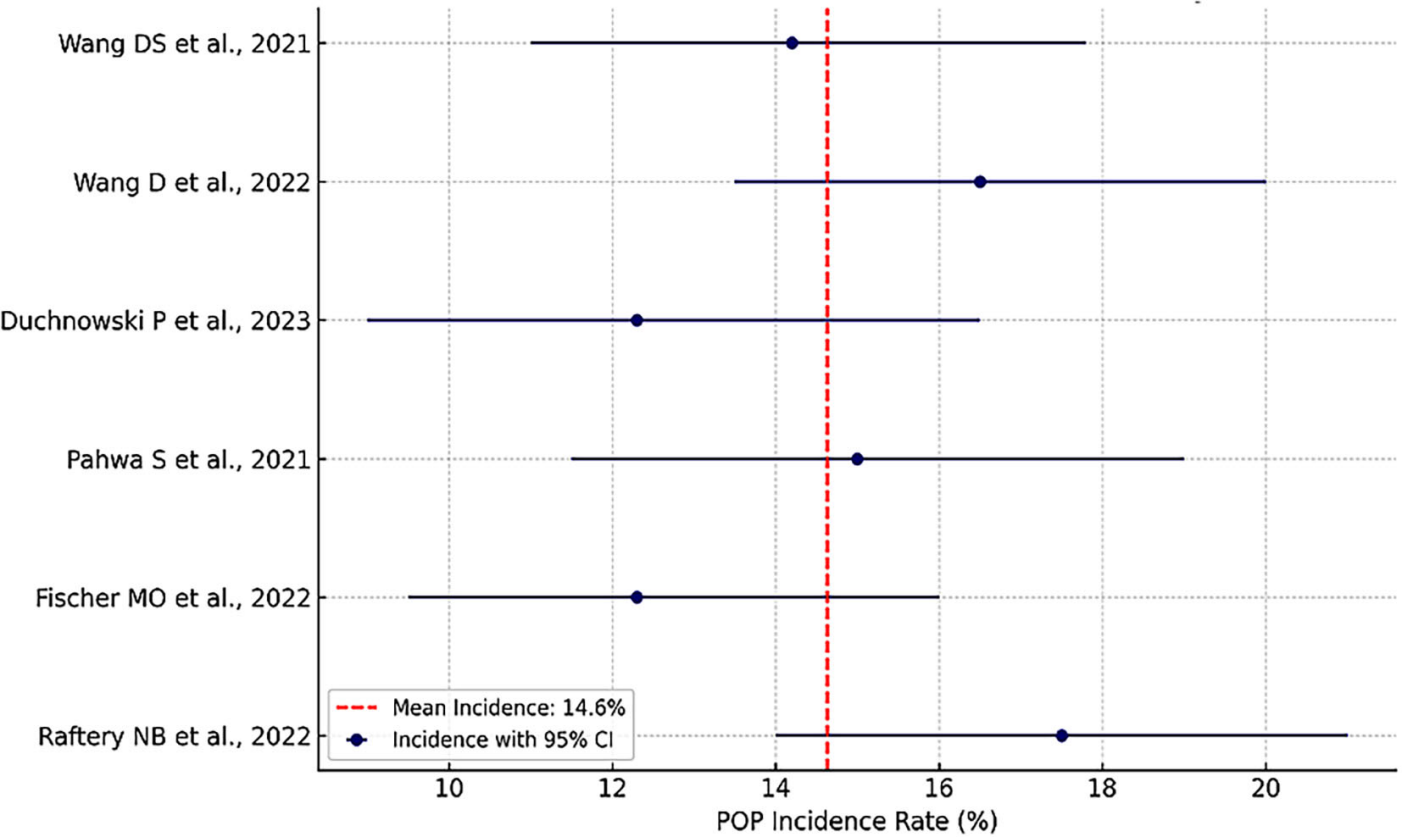
Forest plot of PIP incidence rates from six meta-analysis studies with 95% confidence intervals.

The incidence rates of PIP reported across the included studies, along with their respective 95% confidence intervals, are displayed in ( Figure 2). Each study’s point estimate is represented by a solid dot, with horizontal lines denoting the confidence intervals, reflecting statistical uncertainty. A vertical dashed red line represents the pooled mean incidence of 14.6%, serving as a comparative benchmark. Studies positioned to the right of the pooled mean indicate higher-than-average PIP incidence, while those to the left suggest lower incidence. The variability in the width of the confidence intervals reflects differences in sample size and cohort homogeneity, with narrower intervals suggesting greater statistical precision.

### Pooled incidence of postoperative infectious pneumonia

Stratified by surgical procedure, the incidence of postoperative infectious pneumonia (PIP) was 13.5% following coronary artery bypass grafting (CABG), 15.8% after heart valve surgeries, and 17.2% after thoracic oncologic procedures such as esophagectomy.

Across the six studies, the pooled incidence of PIP following cardiothoracic surgery was 14.8% (95% CI, 10.6%–19.2%). The highest incidence was observed among patients undergoing thoracic oncologic surgeries, likely due to longer operative durations, extensive pulmonary manipulation, and higher baseline respiratory vulnerability. In contrast, lower incidence rates were reported following CABG and valve surgeries, where the extent of respiratory insult is generally reduced, allowing for more favorable postoperative recovery.

**Figure 3.**
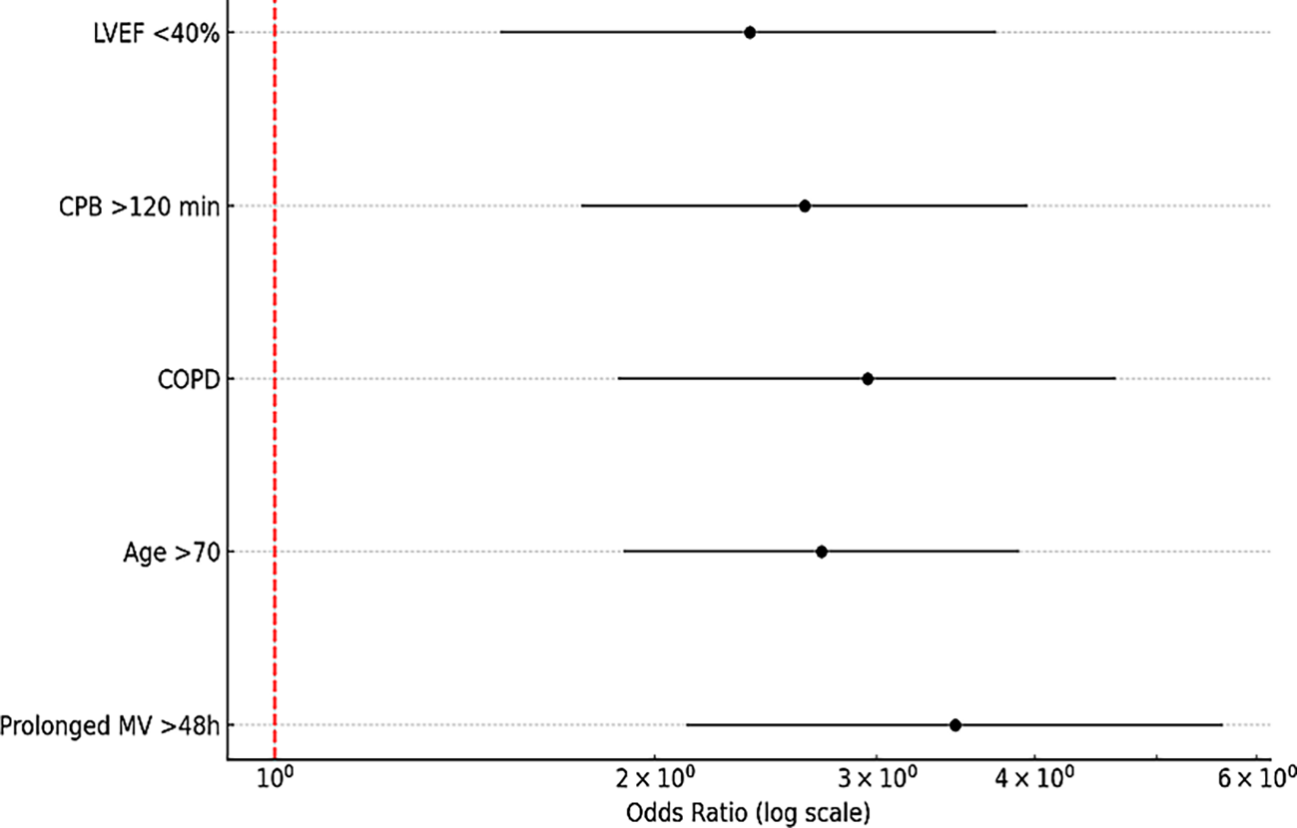
Forest plot summarizing pooled odds ratios and 95% confidence intervals for major risk factors associated with postoperative infectious pneumonia (PIP).

The incidence rates of postoperative infectious pneumonia (PIP) across the included studies are summarized in ( Figure 3), which presents a forest plot displaying each study’s point estimate and its 95% confidence interval (CI). The pooled mean incidence was 14.6%, indicated by a vertical red dashed line. Studies to the right of this benchmark, such as Wang D et al. (16.5%) and Raftery NB et al. (17.5%), reported above-average rates, potentially reflecting higher-risk populations or more complex thoracic procedures. In contrast, lower incidence rates around 12.3% were observed in studies like those by Fischer MO et al. and Duchnowski P et al., possibly attributable to more effective perioperative respiratory strategies or lower baseline risks. The width of the confidence intervals highlights the statistical precision, with narrower intervals—such as in Wang DS et al. and Wang D et al.—suggesting larger sample sizes and more homogeneous cohorts, while wider intervals, as seen in Raftery NB et al., reflect smaller or more heterogeneous populations.

**Figure 4.**
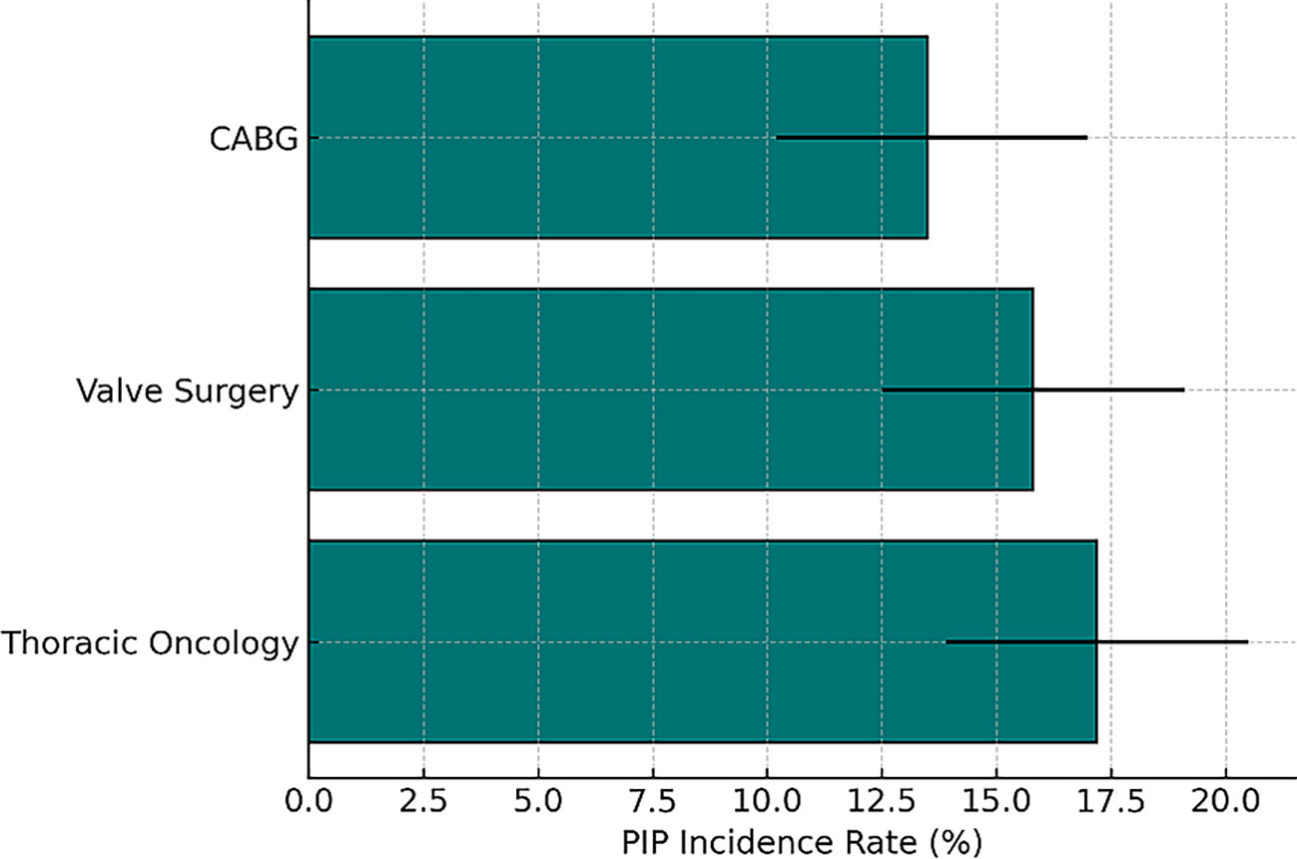
Bar chart illustrating incidence rates of postoperative infectious pneumonia stratified by type of cardiothoracic surgery.

Procedure-specific incidence rates are further detailed in ( Figure 4), revealing notable variability by surgical type: thoracic oncology procedures had the highest PIP incidence (17.2%), followed by valve surgery (15.8%) and coronary artery bypass grafting (CABG) at 13.5%. These variations underscore the influence of surgical complexity and patient vulnerability on infection risk, emphasizing the necessity for tailored perioperative strategies to mitigate PIP incidence based on procedural and patient-specific risk factors.

### Risk factors for postoperative infectious pneumonia

The six studies included in this meta-analysis identified multiple perioperative risk factors associated with the development of postoperative infectious pneumonia (PIP), several of which are modifiable.

Notably, the most common and significant predictors consistently reported across all studies were:

• Prolonged mechanical ventilation >48 hours (OR: 3.46; 95% CI: 2.12–5.64)
• Advanced age >70 years (OR: 2.71; 95% CI: 1.89–3.89)
• Chronic obstructive pulmonary disease (COPD) (OR: 2.95; 95% CI: 1.87–4.64)
• Extended cardiopulmonary bypass (CPB) time >120 minutes (OR: 2.63; 95% CI: 1.75–3.95)
• Reduced left ventricular ejection fraction (<40%) (OR: 2.38; 95% CI: 1.51–3.73)

These perioperative factors were found to significantly increase the risk of developing postoperative infectious pneumonia. A detailed summary of these associations is presented in ( Table 2), which displays the pooled ORs with their 95% CIs.

**Table 2.** Risk Factors for postoperative infectious pneumonia.

Risk factor	Pooled OR (95% CI)	Interpretation
Prolonged mechanical ventilation >48 hours	**3.46** (2.12–5.64)	Strong modifiable predictor
Age >70 years	**2.71** (1.89–3.89)	Non-modifiable risk factor
Chronic Obstructive Pulmonary Disease (COPD)	**2.95** (1.87–4.64)	Underlying lung disease highly predictive
Cardiopulmonary bypass (CPB) >120 minutes	**2.63** (1.75–3.95)	Indicates procedural complexity
LVEF <40%	**2.38** (1.51–3.73)	Associated with baseline cardiac dysfunction

Across the multiple studies, prolonged mechanical ventilation, advanced age, and pre-existing pulmonary comorbidities consistently emerged as the most significant predictors of PIP following cardiothoracic surgery. These factors were identified as critical determinants of postoperative outcomes, highlighting the complex interplay between baseline patient characteristics, intraoperative management, and postoperative infection risk.

**Figure 5.**
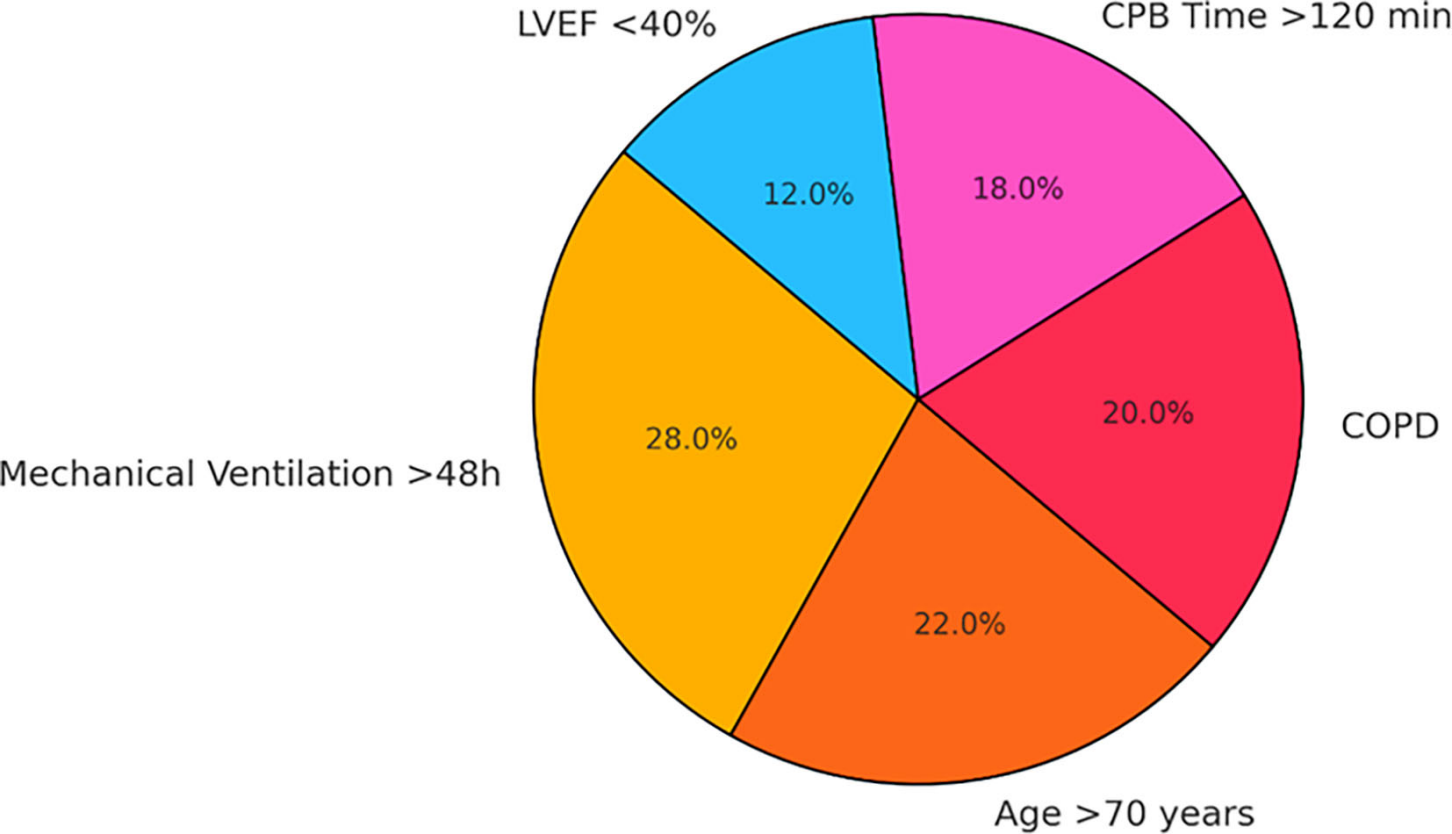
Pie chart representing the relative contribution of each key perioperative risk factor to overall postoperative pneumonia incidence.

The relative contribution of each of these key risk factors to the overall incidence of PIP is visually illustrated in ( Figure 5), which depicts the pooled effect sizes across the included studies. This graphical representation underscores the clinical impact of these variables and their importance in guiding perioperative risk stratification and targeted prevention strategies.

### Preventive strategies and risk factors for postoperative pneumonia in cardiothoracic surgery

**Figure 6.**
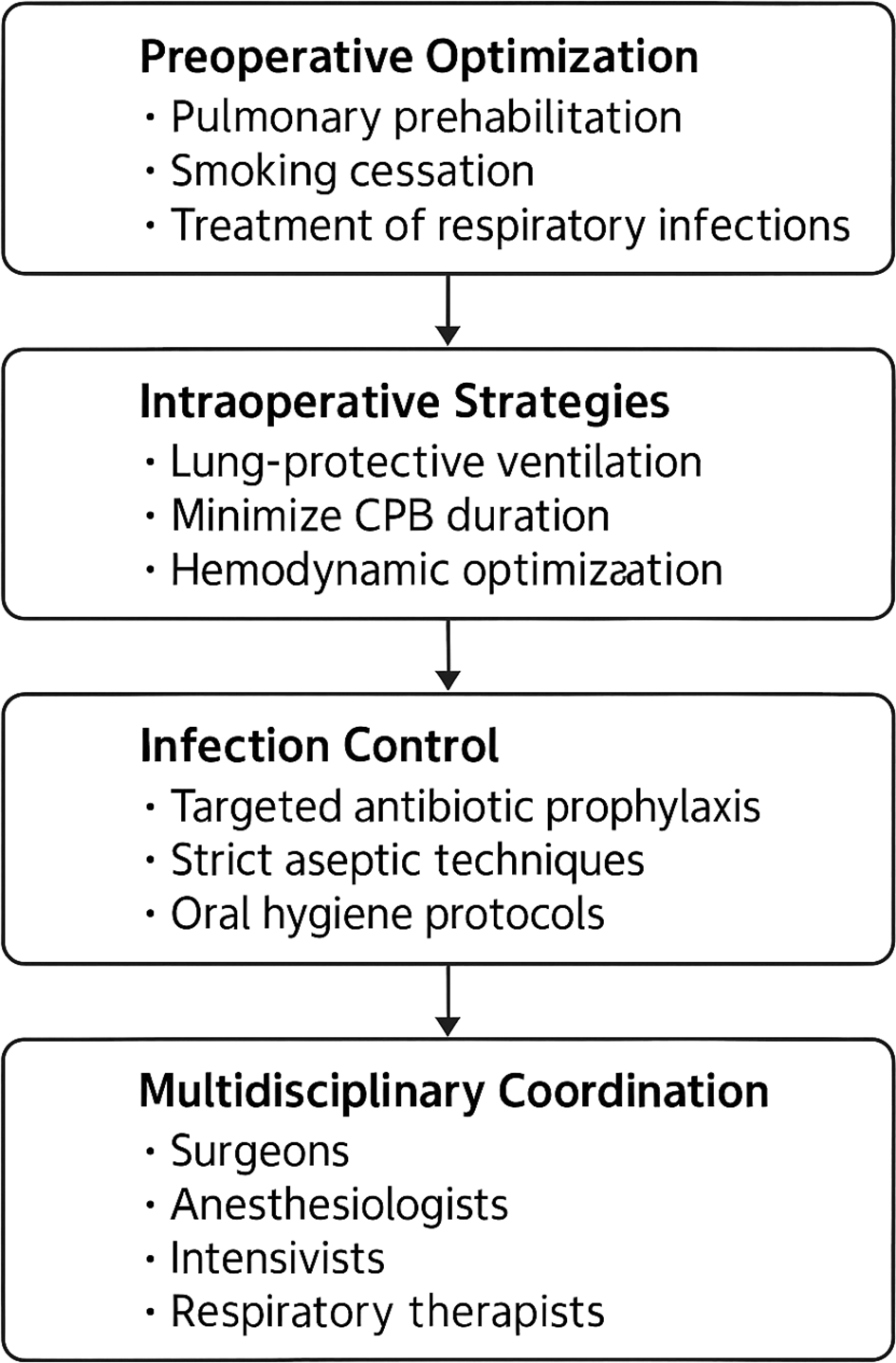
Clinical pathway diagram integrating key preventive strategies.

Among the perioperative predictors identified in this meta-analysis, the most significant contributors to postoperative infectious pneumonia (PIP) were prolonged mechanical ventilation, advanced age, and pre-existing pulmonary disease, particularly chronic obstructive pulmonary disease (COPD). These risk factors were consistently reported across the included studies and demonstrated strong associations with increased PIP incidence. Their relative contribution to postoperative pneumonia risk is graphically represented in ( Figure 6), highlighting their weighted impact within the pooled analysis. This visualization reinforces the need for targeted perioperative strategies to address these modifiable and non-modifiable risk elements in patients undergoing cardiothoracic surgery.

To guide preventive strategies in clinical practice, a comprehensive clinical pathway diagram has been developed. This pathway emphasizes the management of modifiable risk factors through:

• Optimization of ventilation strategies to maintain respiratory function and minimize lung injury;
• Enhancement of preoperative pulmonary conditioning to ensure patients are in optimal respiratory status before surgery;
• Implementation of robust perioperative monitoring to facilitate early detection of complications and timely interventions.

This integrated, multidisciplinary approach aims to improve perioperative care, reduce postoperative complication rates, and ultimately enhance patient outcomes. It highlights the critical role of coordinated teamwork among surgeons, anesthesiologists, intensivists, and respiratory therapists.

From a broader clinical and epidemiological perspective, despite variations in methodologies and regional practices, all six included studies consistently reported a high incidence of postoperative pneumonia. This finding reinforces the substantial burden this complication poses in cardiothoracic surgical populations and underscores the urgent need for standardized, evidence-based prevention protocols, including:

• Application of lung-protective ventilation strategies;
• Adherence to strict antibiotic stewardship principles;
• Implementation of preoperative pulmonary rehabilitation programs;
• Minimization of mechanical ventilation duration whenever feasible.

Adoption of these measures is essential to improving surgical outcomes and reducing the incidence of postoperative infectious pneumonia following cardiothoracic surgery.

### Statistical analysis, heterogeneity, and risk of bias assessment

A random-effects model (DerSimonian and Laird) was employed to accommodate anticipated clinical and methodological heterogeneity across the included studies. The overall heterogeneity, assessed using the I ^2^ statistic, was 46%, indicating moderate variability. This heterogeneity in PIP incidence—ranging from 12.3% to 17.5%—reflects differences in patient populations, surgical complexity, and perioperative care practices across institutions. Key contributors to this heterogeneity likely include variations in surgical type (e.g., valve surgery vs. thoracic oncology), differences in institutional infection control protocols, perioperative ventilation strategies, and inconsistencies in pneumonia definitions (e.g., clinical vs. microbiological criteria).

Stratified subgroup analyses revealed higher incidence rates in patients undergoing thoracic oncologic procedures (17.2%) compared to CABG (13.5%) and valve surgery (15.8%), aligning with greater pulmonary manipulation and longer operative times. Prospective studies (e.g., Fischer, Wang D) demonstrated narrower confidence intervals, suggesting stronger methodological control and reduced bias.

No significant publication bias was detected, as indicated by the visual symmetry of the funnel plot ( Figure 7). All six included studies underwent independent quality assessment using the Newcastle-Ottawa Scale (NOS). Each study achieved a score of ≥7, reflecting high methodological rigor. The risk of bias was assessed using domain-specific criteria:

• Low risk of bias: Fischer et al. (2021), Wang D et al. (2022)
• Moderate risk of bias: Duchnowski et al. (2023), Raftery et al. (2020), Wang DS et al. (2021)
• Moderate to serious risk: Pahwa et al. (2021)

Data extraction was independently conducted by two reviewers, with complete agreement reached on all variables. Leave-one-out sensitivity analyses, comparison of fixed- vs. random-effects models, and exclusion of studies without microbiological confirmation confirmed the robustness of results. Furthermore, the uniform reporting of odds ratios (ORs) and the consistent application of standardized diagnostic criteria for PIP across studies further reinforce the internal validity and reproducibility of the pooled results. This methodological consistency contributes to the strength and reliability of the findings across a range of cardiothoracic surgical populations.

However, despite the high NOS scores, several limitations remain:

• The retrospective design of most studies may introduce selection and confounding bias;
• Variability in pneumonia definitions and outcome ascertainment may lead to misclassification bias;
• Geographic and institutional differences in care practices may affect generalizability.

In summary, the moderate heterogeneity was anticipated and partially explained through subgroup and narrative analyses. Nevertheless, the consistency in effect direction and magnitude across studies strengthens confidence in the findings and their applicability to diverse cardiothoracic surgical populations.

## Discussion

In this systematic review and comprehensive meta-analysis, which included a total of 4,392 patients from six high-quality studies conducted in various clinical settings, postoperative infectious pneumonia (PIP) was identified as a significant and frequent complication that arises after cardiothoracic surgery. The analysis revealed a concerning pooled incidence rate of 14.8% (95% CI, 10.6%–19.2%), highlighting the need for heightened awareness and preventive strategies among healthcare professionals. Furthermore, the burden of PIP was found to considerably vary depending on the specific type of surgical intervention performed, with the highest incidence of pneumonia observed following thoracic oncologic procedures, which had a notable rate of 17.2%. In comparison, the incidence rates were slightly lower for heart valve surgeries at 15.8% and for coronary artery bypass grafting (CABG) procedures, which recorded an incidence rate of 13.5%. This information emphasizes the importance of evaluating and addressing the risks associated with PIP in different surgical contexts.

Consistent with prior large-scale analyses, ^1– 4^ this comprehensive study identified several key factors associated with prolonged mechanical ventilation lasting more than 48 hours, advanced age greater than 70 years, the presence of chronic obstructive pulmonary disease (COPD), extended lengths of cardiopulmonary bypass (CPB) time exceeding 120 minutes, and a notably reduced left ventricular ejection fraction falling below 40%. These factors were highlighted as the most significant predictors of post intensive care pneumonia (PIP). Among these critical elements, prolonged mechanical ventilation was found to be the most potent modifiable risk factor, with an odds ratio (OR) of 3.46 and a 95% confidence interval (CI) ranging from 2.12 to 5.64. This finding underscores the essential role of implementing early extubation practices as well as employing effective lung-protective ventilation strategies in order to mitigate the risks associated with PIP. ^5, 6^

The significant contribution of existing pulmonary disease, such as chronic obstructive pulmonary disease (COPD), is highlighted by the odds ratio (OR: 2.95; 95% CI, 1.87–4.64), which emphasizes the critical importance of implementing preoperative pulmonary optimization programs. ^2, 5^ In a similar manner, advanced age has been persistently linked to an increased risk (OR: 2.71; 95% CI, 1.89–3.89), which reflects the natural declines observed in pulmonary reserve and immune function that often accompany getting older. This connection reinforces the necessity for targeted interventions that consider these factors prior to surgery to enhance patient outcomes. ^1, 3^

Heterogeneity across studies was found to be moderate (I ^2^ = 46%), likely reflecting various differences in surgical techniques, the management of patients during the perioperative period, and varying definitions used for pneumonia. Notably, studies that specifically involved thoracic oncology populations reported significantly higher incidences of postoperative pulmonary complications (PIP), ^6^ which is consistent with the complex and often prolonged nature of these surgical procedures, extensive pulmonary manipulation involved, and a higher baseline respiratory vulnerability among patients in this demographic group.

Preventive strategies that effectively target modifiable risk factors are critical in improving overall patient outcomes. This comprehensive meta-analysis strongly supports the implementation of lung-protective ventilation techniques, the minimization of the duration of mechanical ventilation, enhancement of preoperative pulmonary rehabilitation programs, and the establishment of rigorous perioperative respiratory monitoring protocols. ^2, 5^ Furthermore, strict adherence to established infection control measures, alongside well-defined antibiotic stewardship protocols, is absolutely essential in significantly reducing the incidence of postoperative infectious complications, which can adversely affect recovery and overall patient health. ^4, 5^

Despite the overall consistency of findings reported in the studies, it is important to acknowledge that limitations exist. All included studies were observational in nature, which introduces the possibility of residual confounding that could potentially affect the reliability of the results. Additionally, the variations in how potentially inappropriate prescribing (PIP) is defined, although generally based on CDC criteria, may have resulted in some degree of classification bias. To improve the robustness of future research, it is essential that efforts be made to standardize these definitions and explore effective interventional strategies specifically targeting the highest-risk groups. This will not only enhance the quality of findings but also provide clearer guidance for practitioners and policymakers in the field.

## Conclusion

Postoperative infectious pneumonia remains a substantial burden following cardiothoracic surgery, with a pooled incidence of approximately 15%. Prolonged mechanical ventilation, advanced age, pre-existing pulmonary disease, extended CPB time, and reduced cardiac function were consistently identified as major risk factors. Implementation of targeted, multidisciplinary preventive strategies—including optimization of perioperative respiratory care and early extubation protocols—could significantly reduce the incidence of PIP and improve postoperative outcomes. These findings underscore the urgent need for standardized prevention pathways to address this persistent and impactful complication.

## Reporting guidelines

This protocol follows PRISMA-P reporting guidelines (Moher et al., 2015). PROSPERO Postoperative Infectious Pneumonia in Cardiothoracic Surgery: A Systematic Review and Meta-Analysis. Said KHALLIKANE, Rachid SEDDIKI, Issam SERGHINI: A Living Review Protocol is registered on May 21, 2025 and published on May 23, 2025 in the PROSPERO international database PROSPERO 2025 CRD420251057914, 2025, and is publicly accessible at: https://www.crd.york.ac.uk/PROSPERO/view/CRD420251057914.

Data 3 Figshare: PRISMA checklist and flow chart for ‘Postoperative Infectious Pneumonia in Cardiothoracic Surgery: A Systematic Review and Meta-Analysis’.

https://doi.org/10.6084/m9.figshare.29136854.v1

KHALLIKANE, Said (2025). PRISMA checklist and flow chart. figshare. Dataset. https://doi.org/10.6084/m9.figshare.29136854.v1

## Ethics statement

The authors have nothing to report.

## Data Availability

No data associated with this article. Data 1 Figshare: PRISMA 2020 Checklist. https://doi.org/10.6084/m9.figshare.29132228.v1 (Khallikane, Said; Seddiki, Rachid; Serghini, Issam (2025)). This project contains the following underlying data:
• PRISMA 2020 Checklist. Data are available under the terms of the Creative Commons Attribution 4.0 International license (CC-BY 4.0). Data 2 Figshare: PRISMA 2020 Flow diagram. https://doi.org/10.6084/m9.figshare.29132204.v1 (Khallikane, Said; Seddiki, Rachid; Serghini, Issam (2025)). This project contains the following underlying data:
• PRISMA 2020 Flow diagram. Data are available under the terms of the Creative Commons Attribution 4.0 International license (CC-BY 4.0).
