# Supplementary material for: Postoperative Infectious Pneumonia in Cardiothoracic Surgery: A Systematic Review and Meta-Analysis
Source: F1000Res. 2025 Jun 16;14:588. [Version 1] doi: 10.12688/f1000research.165457.1 (PMC12268255; doi:10.12688/f1000research.165457.1)
Supplement: Peer review history

# Reviewer report: Reviewer response for version 1

**DOI:** 10.5256/f1000research.182096.r398105

**Published:** 2025-07-16

**Authors:** Bastiaan M Gerritse

**Affiliations:** 1 Amphia Hospital, Molengracht, Netherlands Antilles

I would like to recommend the authors of this very well written systematic review and meta-analysis: this is how is should be done! I have no questions or remarks for improvement of this publication. I hope this publication will be an incentive for the improvement of medical care of cardio-thoracic surgery patients.

Are the rationale for, and objectives of, the Systematic Review clearly stated?

Yes

Is the statistical analysis and its interpretation appropriate?

Yes

If this is a Living Systematic Review, is the ‘living’ method appropriate and is the search schedule clearly defined and justified? (‘Living Systematic Review’ or a variation of this term should be included in the title.)

Yes

Are sufficient details of the methods and analysis provided to allow replication by others?

Yes

Are the conclusions drawn adequately supported by the results presented in the review?

Yes

Reviewer Expertise:

Cardiothoracic Anesthesia, Intensive Care and Emergency Medicine

I confirm that I have read this submission and believe that I have an appropriate level of expertise to confirm that it is of an acceptable scientific standard.

# Reviewer report: Reviewer response for version 1

**DOI:** 10.5256/f1000research.182096.r392908

**Published:** 2025-06-25

**Authors:** Chlouchi Abdellatif

**Affiliations:** 1 Military Hospital Mohammed, Rabat, Morocco

This is a well-conducted and clearly written systematic review and meta-analysis on a clinically relevant issue: postoperative infectious pneumonia after cardiothoracic surgery. The methodology is solid, the research question is clearly defined, and the statistical approach is appropriate. The authors followed PRISMA 2020 guidelines, registered their protocol in PROSPERO, and included only high-guality cohort studies. Data collection, risk of bias assessment, and outcome reporting are all transparent and well presented The findings are meaningful: the reported incidence of PIP is consistent with previous literature, and the identified risk factors particularly prolonged ventilation and pre-existing lung disease are clinically important.

Are the rationale for, and objectives of, the Systematic Review clearly stated?

Yes

Is the statistical analysis and its interpretation appropriate?

Yes

If this is a Living Systematic Review, is the ‘living’ method appropriate and is the search schedule clearly defined and justified? (‘Living Systematic Review’ or a variation of this term should be included in the title.)

Yes

Are sufficient details of the methods and analysis provided to allow replication by others?

Yes

Are the conclusions drawn adequately supported by the results presented in the review?

Yes

Reviewer Expertise:

In Intensive care medicine, after cardiac sergery, infectious disease is a major complication.

I confirm that I have read this submission and believe that I have an appropriate level of expertise to confirm that it is of an acceptable scientific standard.
